# Three-port laparoscopic cholecystectomy with common bile duct exploration in situs inverse totalis: a case report and literature review

**DOI:** 10.3389/fsurg.2026.1775353

**Published:** 2026-06-16

**Authors:** Cuinan Jiang, Yang Yang, Kang Zhao, Jichao Tang, Huan Gu, Yi Li

**Affiliations:** Section for Day Surgery, Department of General Surgery, The Third People's Hospital of Chengdu & The Affiliated Hospital of Southwest Jiaotong University, Chengdu, China

**Keywords:** case report, cholelithiasis, laparoscopic common bile duct exploration, primary suture, situs inverse totalis

## Abstract

**Introduction:**

The anatomical variations in patients with situs inverse totalis (SIT) pose unique challenges to laparoscopic cholecystectomy with common bile duct exploration (LC + LCBDE) that relies on conventional anatomical memory.

**Case presentation:**

In this case report, we present a 59-year-old female patient who presented with a 2-year history of recurrent upper abdominal pain that had worsened over the preceding 5 days. After evaluation, she was diagnosed to be a case of SIT with cholelithiasis, obstructive jaundice, acute biliary pancreatitis and atrial fibrillation. She subsequently underwent LC + LCBDE. The procedure was performed using a three-port technique: the 5-mm trocar port at the subxiphoid site was primarily used for gallbladder retraction and also served as the access path for choledochoscopic exploration, while the 10-mm port in the left lumbar region was mainly utilized for resection maneuvers and functioned as the channel for the clip applier. During the operation, silt-like stones in the bile duct were irrigated and cleared under choledochoscopic guidance, followed by primary closure of the choledochotomy. The patient's postoperative recovery was uneventful, and discharge was achieved on the second postoperative day.

**Conclusion:**

This case highlights the technical challenges of performing laparoscopic biliary surgery in a patient with SIT. The strategic placement of trocar ports was crucial for minimizing surgical trauma and ensuring procedural safety. Furthermore, given the patient's comorbidity of atrial fibrillation, our management demonstrates that meticulous perioperative preparation and a tailored surgical strategy can facilitate enhanced recovery in complex SIT cases.

## 1 Introduction

Laparoscopic cholecystectomy (LC) and laparoscopic common bile duct exploration (LCBDE) is a commonly used minimally invasive approach for cholecystolithiasis and choledocholithiasis. This procedure offers more intuitive and precise advantages, effectively preserving the function of the patient's sphincter of Oddi and reducing the incidence of long-term complications (1, 2).

Situs inversus totalis (SIT) is a rare congenital anatomical anomaly, first described by Fabricius in 1,600 and has a reported incidence of between 1:8,000 to 1:20,000 (3, 4). It is characterized by a complete mirror-image symmetry of all major organs within the thoracic and abdominal cavities compared to the normal anatomical arrangement. Situs inversus can be further subdivided into SIT, where both the cardiac position and abdominal viscera are completely reversed (mirror-image dextrocardia), and situs inversus with levocardia, a rare variant where the heart remains on the left side despite reversal of the abdominal organs (5).

The embryological basis of situs inversus involves abnormal left-right axis determination during early embryonic development. Normal lateralization is established during gastrulation through a complex cascade involving the node, a transient embryonic structure that generates leftward fluid flow via motile monocilia (6). This nodal flow activates asymmetric expression of key signaling molecules, including Sonic Hedgehog (Shh), Nodal, and Lefty proteins, which ultimately lead to left-sided expression of the transcription factor PITX2 (7). Mutations affecting ciliary structure or function disrupt this left-right patterning cascade, resulting in randomization or complete reversal of organ laterality (6, 7). Situs inversus is frequently associated with primary ciliary dyskinesia (PCD), a genetic disorder characterized by defective motile cilia affecting approximately 1 in 12,500–1 in 50,000 individuals (8). Approximately 50% of patients with PCD exhibit situs inversus, a combination known as Kartagener syndrome, which comprises the classic triad of situs inversus, chronic sinusitis, and bronchiectasis (8).

Although the incidence of biliary tract diseases in patients with SIT is comparable to that in the general population, the mirror-image anatomy presents significant challenges for surgery (9). This is mainly manifested in the disruption of conventional thinking patterns, visual inversion, and operational inertia. In this case, we report a SIT patient with cholelithiasis, obstructive jaundice, acute biliary pancreatitis and atrial fibrillation managed with LC + LCBDE and provide a brief review of the relevant literature. This case report was prepared following the CARE guidelines (10).

## 2 Case presentation

### 2.1 History and physical examination

A 59-year-old female patient was admitted to our hospital with a chief complaint of recurrent upper abdominal pain for 2 years, which had worsened over the past 5 days and was accompanied by vomiting of gastric contents. The patient had a history of atrial fibrillation for over 30 years and has been taking rivaroxaban regularly. The patient's family and psychological histories were unremarkable, and there were no prior interventions related to the current condition before this episode. Physical examination revealed mild scleral icterus and epigastric tenderness, without evidence of rebound tenderness, guarding, organomegaly, abdominal masses, or ascites (no shifting dullness).

### 2.2 Auxiliary examinations

The preoperative complete blood count revealed an elevated neutrophil percentage of 86.6% (reference range, 40%–75%). Liver function tests showed a total bilirubin level of 61.9 μmol/L (reference value, <28 μmol/L), predominantly due to an elevated direct bilirubin level of 44.8 μmol/L (reference value, <9 μmol/L). Concurrently, the alanine aminotransferase (ALT) level was 448 U/L (reference value, <40 U/L) and the aspartate aminotransferase (AST) level was 217 U/L (reference value, <40 U/L). Serum amylase was significantly elevated at 2,564.7 U/L (reference range, 25–104 U/L). Other laboratory parameters, including coagulation profile, renal function, electrolytes, lipid panel, and cardiac markers, were all within normal limits.

The upper abdominal magnetic resonance imaging (MRI) plain scan revealed complete situs inversus (Figure 1A). Magnetic resonance cholangiopancreatography (MRCP) showed several nodular filling defects within the gallbladder lumen (Figure 1B). Computed tomography (CT) findings indicated slight pancreatic swelling with blurring of the peripancreatic fat planes and the presence of strand-like opacities (Figure 1C).

**Figure 1:**
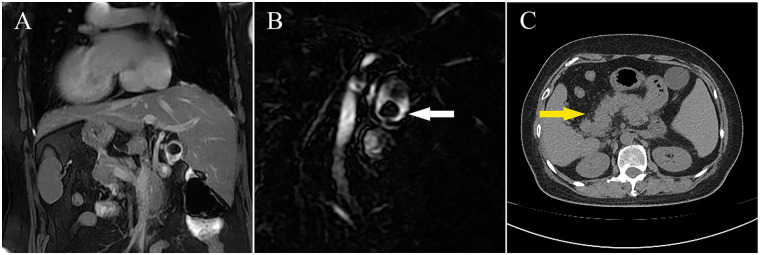
Preoperative imaging findings. **(A)** Magnetic resonance imaging (MRI) scan showing situs inversus totalis (SIT). **(B)** Magnetic resonance cholangiopancreatography (MRCP) revealing multiple gallstones within the gallbladder (white arrow). **(C)** Computed tomography (CT) scan demonstrating the feature indicative of acute pancreatitis (yellow arrow).

Ambulatory electrocardiographic monitoring over 24 h demonstrated atrial fibrillation, during which the maximum heart rate attained was 168 beats per minute (bpm).

### 2.3 Preoperative diagnosis and preoperative preparation

Based on the patient's medical history and auxiliary examinations, the patient was initially diagnosed with cholelithiasis, acute biliary pancreatitis, obstructive jaundice, atrial fibrillation, and SIT. According to the Revised Atlanta Classification and Definitions, the patient was classified as having mild acute pancreatitis of the interstitial edematous pancreatitis type. The patient exhibited no organ failure, with a modified Marshall score of 0 (11, 12). The treatment regimen included nothing by mouth (NPO), fluid resuscitation, anti-infective therapy, somatostatin (3 mg every 12 h via continuous infusion), and gabexate mesylate injection (300 mg once daily via intravenous infusion). The patient developed a rapid ventricular rate due to atrial fibrillation. Metoprolol sustained-release tablets were administered orally at a dose of 47.5 mg once daily, following which the ventricular rate was controlled within 80–90 bpm. Concurrently, the administration of rivaroxaban was halted 48 h prior to surgery and received bridging with enoxaparin 4,000 AxaIU subcutaneously once daily. According to the Charlson Comorbidity Index (CCI) score, the patient's comorbidity burden was classified as low risk (score 0) (13). Additionally, based on the Tokyo Guidelines 2,018 classification, the severity of cholecystitis was graded as Grade I (mild) (14). The patient was evaluated preoperatively by the anesthesiologist and was classified as American Society of Anesthesiologists (ASA) physical status II with New York Heart Association (NYHA) functional class II.

### 2.4 Surgical technique

Under general anesthesia, the patient was positioned supine with a 20–30° reverse Trendelenburg tilt and a 15° right lateral tilt to facilitate exposure of the left-sided gallbladder and allow gravitational displacement of the bowel loops away from the operative field. The operating room setup was arranged in a mirror-image configuration to accommodate the reversed anatomy. The laparoscopic stack, monitors, and CO2 insufflator were positioned at the patient's left shoulder. However, we still recommend placing a monitor near the patient's right shoulder to facilitate observation by the assistant (Figure 2). The primary surgeon stood on the patient's right side, and the assistant surgeon stood on the patient's left side.

**Figure 2:**
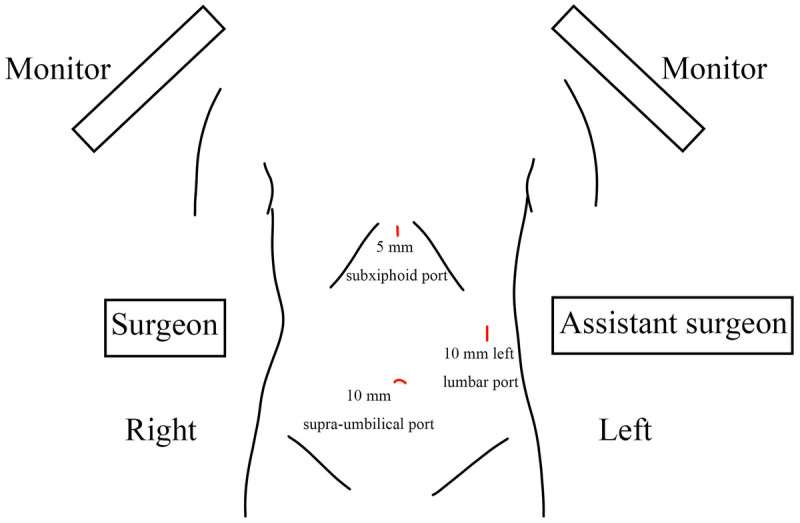
Schematic diagram of the port placement configuration and the positions of the surgical team. The 5-mm subxiphoid port was primarily used for retraction, exposure, and insertion of the choledochoscope. The 10-mm left lumbar port was primarily used for operating and introducing the clip applier. The 10-mm supra-umbilical port was used for the laparoscope.

The port placement is illustrated in Figure 2. A 10-mm port was inserted supra-umbilically to serve as the laparoscopic observation port and for connection to the CO_2_ pneumoperitoneum tube. Under direct laparoscopic vision, a 5-mm operating port was placed subxiphoid, which also served as the access port for choledochoscopic exploration. A 10-mm operating port was positioned in the left lumbar region, which also functioned as the entry port for the clip applier.

The adhesions within the abdominal cavity were dissected using the electrosurgical knife, exposing the gallbladder (Figures 3A, B). The Calot's triangle was carefully anatomized to identify the cystic duct and cystic artery, which were then clipped with absorbable clips and transected (Figure 3C). The gallbladder was subsequently dissected from the liver bed. A longitudinal incision approximately 5–7 mm in length was made on the anterior wall of the common bile duct (CBD), just inferior to the cystic duct stump. A 3.5-mm choledochoscope was introduced through the 5-mm subxiphoid port to explore the biliary tract (Figure 3D). Exploration with the choledochoscope revealed the presence of silt-like stones within the CBD. Manual compression of the saline bag connected to the instrument channel accelerates the irrigation flow rate, thereby facilitating the effective lavage of silt-like stones from the biliary tract. Choledochoscopic examination revealed no significant congestion or edema at the distal end of the CBD, and the scope was advanced successfully through the duodenal papilla into the duodenum. Subsequently, the choledochotomy was closed with interrupted sutures using a 4–0 PDP suture (Figures 3E, F). The choledochotomy closure was performed with the surgeon's dominant hand through the left lumbar port, which provided optimal ergonomic positioning for precise suturing in this unfamiliar anatomical configuration. The gallbladder was then placed into a retrieval bag and extracted through the left lumbar port. A 16 Fr abdominal drainage tube was placed in the subhepatic space.

**Figure 3:**
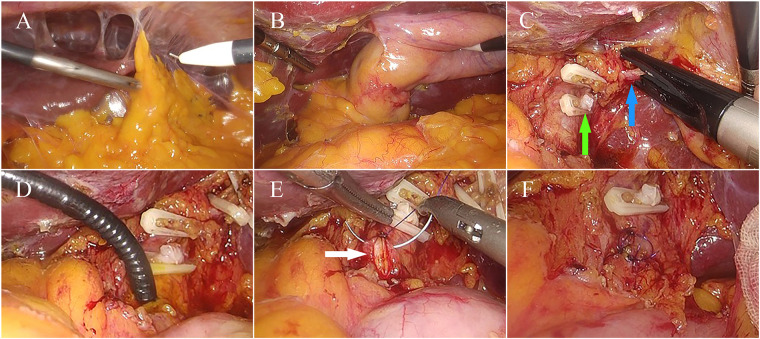
Intraoperative findings of surgical procedure. **(A)** Separation of intra-abdominal adhesions. **(B)** The gallbladder is situated on the left side due to situs inversus. **(C)** The cystic duct and cystic artery were identified and divided (green arrow: the residual end of the cystic duct, blue arrow: the cystic artery). **(D)** Choledochoscope exploration. **(E,F)** Primary closure of choledochotomy with 4-0 PDP sutures (white arrow: choledochotomy).

### 2.5 Postoperative management

Only mild surgical incision pain was reported by the patient, with no additional adverse effects noted. Liquid diet intake was commenced postoperatively. The patient gradually resumed a normal diet on the first postoperative day (POD 1), and the results of follow-up hematological examinations (complete blood count, liver/renal function, and serum amylase) were unremarkable. On POD 2, approximately 5 mL of light bloody fluid was drained, following which the abdominal drain was removed and the patient was discharged uneventfully. The patient resumed oral rivaroxaban at 48 h postoperatively and was scheduled for regular follow-up at the cardiology clinic.

### 2.6 Follow up

One month after the operation, the patient presented for a scheduled follow-up. She denied any specific discomfort and had resumed a normal diet. Physical examination revealed a well-healed surgical incision. Results of the blood tests (complete blood count and liver function tests) and abdominal ultrasound were all within normal limits. Abdominal ultrasound revealed no significant abnormalities, with no evident ascites and no significant dilatation of the intra- or extrahepatic bile ducts.

## 3 Discussion

The concept of a 'single-stage procedure' combining LC and LCBDE is advocated by many surgeons globally for the management of patients with gallstones combined with CBD stones. LCBDE can reduce the reliance on preoperative imaging resources, such as MRCP, by utilizing the direct visualization offered by intraoperative choledochoscopy (15).

In patients with SIT, the visceral organs are mirrored from their usual positions. LC with or without LCBDE presents unique technical challenges due to the reversed anatomy. In this unfamiliar surgical landscape, surgeons must exercise particular caution to avoid inadvertent injury. In our literature review, we identified a total of 11 reported cases of patients with SIT who underwent LC + LCBDE for the treatment of cholecystolithiasis and choledocholithiasis. In one case, the surgeon operated between the patient's legs, while in the majority of other cases, the surgeon stood on the right side of the patient (16). Regarding the port arrangement, the “mirrored American approach” was employed in three cases (17–19), while the “mirrored French approach” was used in one case (20). In two reports, the “mirrored conventional approach” described by the authors could refer to either the mirrored American or French approach (16, 21). One case utilized the “modified mirrored American approach”, which applied an additional right pararectal port for common bile duct exploration (CBDE) (22). Another case adopted a “left-side port strategy” due to the presence of dense adhesions in the midline of the abdominal cavity, confining the surgical maneuvers to the left abdominal quadrant (23). Other operative details and postoperative outcomes are presented in Table 1.

**Table 1:** Preoperative and intraoperative parameters of the patients with situs inversus totalis treated by laparoscopic common bile duct exploration in the literature review.

Author/year	Age/ sex	Preoperative ERCP	Style	Incision number	Surgeon's position	Working hand	IOC or Choledochoscope	CBDE technique	Postoperative hospital stay (days)
Tai et al, 2004 (24)	NM/F	Yes	NM	4	NM	NM	NM	NM	NM
Kang et al, 2004 (17)	64/F	No	Mir-A	4	Right side	Left	Choledochoscope	T tube drainage following choledochotomy	NM
Han et al, 2012 (21)	71/F	Yes	Mir-C	4	NM	NM	Choledochoscope	Choledochotomy	5
Liu et al, 2017 (25)	51/F	No	NM	NM	NM	NM	NM	NM	7
Senthilnathan et al, 2017 (16)	76/F	Yes	Mir-C	4	Between legs	NM	IOC	LCDD following choledochotomy	7
Takalkar et al, 2018 (18)	50/F	Yes	Mir-A	4	NM	NM	NM	LCDD following choledochotomy	NM
Simkhada et al, 2021 (19)	63/F	Yes	Mir-A	4	Right side	Left	Choledochoscope	Primary closure following choledochotomy	5
Chiu et al, 2023 (26)	79/F	No	SP	2	Right side	Right	IOC	Transcystic approach with “basket-in-catheter"	3
Matsuura et al, 2024 (20)	78/F	Yes	Mir-F	4	Right side	Right	Choledochoscope	Transcystic approach with C-tube drainage	NM
Krishna et al, 2025 (22)	56/F	Yes	M-mir-A	4	Right side	Left	Choledochoscope	Primary closure following choledochotomy	3
Nagata et al, 2025 (23)	70/F	Yes	LSP	4	NM	NM	Choledochoscope	Primary closure following choledochotomy	8
Our case	59/F	No	Mir-A	3	Right side	Right	Choledochoscope	Primary closure following choledochotomy	2

F, female; NM, not mentioned; Mir-A, mirrored American; Mir-F, mirrored French; Mir-C, mirrored conventional; M-mir-A, modified mirrored American; SP, single-port; LSP, left-side-port; IOC, intraoperative cholangiography; LCDD, laparoscopic choledochoduodenostomy.

In our case, we employed a three-port technique for the procedure. A cohort study found that compared to the standard multi-port approach, single-incision LC is associated with a statistically significantly higher long-term incisional hernia rate at the umbilical port site. Moreover, there was no relevant advantage regarding the postoperative course, hospital stay or cosmetic satisfaction (27). Compared with the four-port approach, the three-port approach can effectively reduce surgical trauma. A comprehensive systematic review and meta-analysis encompassing 18 randomized controlled trials with 2,085 patients, provided further evidence supporting the three-port approach (28). The analysis demonstrated that three-port LC was associated with shorter hospital stay (*p* < 0.001) and reduced postoperative analgesia requirements (*p* < 0.001) compared to the four-port technique. Importantly, no significant differences were observed in operative time, procedure success rate, or bile duct injury rates between the two approaches, suggesting that the three-port technique does not compromise safety while offering tangible patient benefits. Therefore, the three-port approach represents a suitable option. However, critics argue that the three-port approach may present technical challenges, particularly in complex cases requiring CBDE. The reduced number of working ports necessitates greater instrument manipulation and may limit the surgeon's ability to achieve optimal exposure in difficult anatomical situations. In the context of SIT, where the reversed anatomy already imposes cognitive and technical demands, the decision to employ a three-port approach requires careful consideration of the surgeon's experience and the specific anatomical challenges presented by the individual patient.

To minimize the risk of inadvertent injury attributable to the surgeon's unfamiliarity to the reversed anatomy in SIT patients and to account for the predominance of right-handedness among surgeons, the trocar arrangement is of special importance. Accordingly, we implemented a port strategy similar to the “mirrored American approach”. The surgeon's left hand, via a 5-mm trocar port at the subxiphoid site, was used to retract the gallbladder and expose the surgical field, while the right hand performed the dissection through a 10-mm port in the left lumbar region. This setup not only accommodates the ergonomics of a right-handed surgeon but also allows for cooperation of switching hands when needed. The 5-mm subxiphoid trocar port also served for CBDE. This approach significantly shortened the path required for the choledochoscope to reach the CBD. Moreover, as the subxiphoid port is situated directly above the choledochotomy incision, it facilitated smooth entry of the scope. Additionally, if T-tube drainage was indicated, the T-tube could be externalized directly through this same subxiphoid port.

Although preoperative MRCP did not indicate definitive stones in the bile duct, the patient presented with concomitant acute pancreatitis and obstructive jaundice. Therefore, we prudently proceeded with intraoperative CBDE. Under the direct visualization of choledochoscopy, silt-like stones were found remaining in the bile duct, which could potentially affect the patient's postoperative recovery. Given that a significant proportion of patients with CBD stones develop symptoms of obstructive jaundice, cholangitis, and pancreatitis during acute episodes, performing bile duct exploration concomitant with cholecystectomy may be beneficial. This combined approach can help avoid retained CBD stones after cholecystectomy and prevent readmission, thereby reducing the patient's medical burden and healthcare costs (29).

The management of the choledochotomy following LCBDE represents another critical decision point with significant implications for patient outcomes. The traditional approach of T-tube drainage, while providing external biliary decompression and a route for postoperative cholangiography, has been increasingly challenged by evidence supporting primary duct closure (PDC). In our case, the patient presented with no signs of inflammation or edema in the CBD wall, no residual stones, and no obstruction at the distal end of the CBD, while mild dilation of the CBD was observed. Therefore, PDC was deemed appropriate. Compared with T-tube drainage, PDC is associated with shorter operative time, shorter postoperative hospital stay, and shorter duration of abdominal drainage (30, 31). It also avoids the inconveniences and complications associated with long-term indwelling T-tube after discharge, thus contributing to a better postoperative recovery for the patient (32). Notably, the patient was discharged only two days postoperatively, achieving the shortest hospital stay in all reported cases.

## 4 Conclusion

The mirror-image anatomy presents considerable challenges for LC + LCBDE in SIT patients. An optimal trocar arrangement, however, is key to reducing surgical trauma while enabling the procedure to approximate the conventional operative habits. Additionally, thorough perioperative preparation and a tailored surgical strategy can shorten postoperative hospitalization and promote rapid recovery in SIT patients with complex conditions.

## Data Availability

The raw data supporting the conclusions of this article will be made available by the authors, without undue reservation.
